# Who Should Engage in the Practice of Surgery

**Published:** 1914-03

**Authors:** E. C. Davis

**Affiliations:** Atlanta, Ga.


### Journal-Record of Medicine

Successor to Atlanta Medical and Surgical Journal, Established 1855
and Southern Medical Record, Established 1870

**OWNED BY THE ATLANTA MEDICAL JOURNAL COMPANY**

PUBLISHED MONTHLY

*Official Organ Fulton County Medical Society, State Examining Board, Presbyterian Hospital, Atlanta, Birmingham and Atlantic Railroad Surgeons' Association, Chattahoochee Valley Medical and Surgical Association, Etc.*

EDGAR BALLENGER, M. D., Editor
BERNARD WOLFF, M. D., Supervising Editor
A. W. STIRLING, M. D., C. M., D. P. H.; J. S. HURT, B. Ph., M.D.
GEO. M. NILES, M. D., W. J. LOVE, M. D., (Ala.); Associate Editors
E. W. ALLEN, Business Manager

**COLLABORATORS**

DR. W. F. WESTMORLAND, General Surgery
F. W. McRAE, M. D., Abdominal Surgery
H. F. HARRIS, M. D., Pathology and Bacteriology
E. B. BLOCK, M. D., Diseases of the Nervous System
MICHAEL HOKE, M. D., Orthopedic Surgery
CYRUS W. STRICKLER, M. D., Legal Medicine and Medical Legislation
E. C. DAVIS, A. B., M. D., Obstetrics
E. G. JONES, A. B., M. D., Gynecology
R. T. DORSEY, Jr., B. S., M. D., Medicine
L. M. GAINES, A. B., M. D., Internal Medicine
GEO. C. MIZELL, M. D., Diseases of the Stomach and Intestines
L. B. CLARKE, M. D., Pediatrics
EDGAR PAULIN, M. D., Opsonic Medicine
THEODORE TOEPEL, M. D., Mechano Therapy
R. R. DALY, M. D., Medical Society
A. W. STIRLING, M. D., Etc., Diseases of the Eye, Ear, Nose and Throat
BERNARD WOLFF, M. D., Diseases of the Skin
E. G. BALLENGER, M. D., Diseases of the Genito-Urinary Organs

VOL. LX. ATLANTA, GA., MARCH, 1914. No. 12

### WHO SHOULD ENGAGE IN THE PRACTICE OF SURGERY.

BY E. C. DAVIS A. B., M. D., ATLANTA, GA

The selection of such a subject for a paper before this Society may be construed by many as rather presumptive on my part and render me liable to severe criticism by some of my most esteemed friends; but the interest which the profession, and especially the public, have recently given to this has caused me to be willing to run this risk of censure, in order that my views may be expressed.

Many may disagree with me throughout, but in so doing, I only request that the commercial side of this question be omitted and the scientific and humane alone allowed to enter into the discussion.

When the practice of surgery with limitations is alluded to, we only include major surgery and not the simple or minor surgical care or emergency surgery which must be attended to at once to save life or avoid infections.

No field of life offers more fruitful opportunities for deception and fraud than does surgery of today. When the work of the surgeon was considered only after all other means had been exhausted, then the patient or family could easily decide that nothing irregular was being practiced, but today when laboratory methods and pathological reports may be made, then it is a fruitful field for mistakes or dishonest practices if those engaged are not of the right training or development. The surgical errors are usually of such a serious nature prolonging convalescene, permanent impairment of function of some organ or set of organs, or the death of the patient. If such possibilities as these are liable, then it goes without saying that he who engages in such responsibilities should enter upon them with the very best possible equipment. The best methods of attaining these results is the intention of this paper.

In order that one should be properly equipped for practice of surgery today, the preliminary preparations for the study of medicine should be very thorough. In fact, the time is not very far distant when a collegiate degree or its equivalent should be required before one is allowed to enter upon the proper study of medicine. If this equipment is provided for then the study of medicine should consume the usual four years with special attention being devoted to anatomy, physiology, diagnosis and pathology.

I would like especially to emphasize the importance of a thorough knowledge of physiology as upon it hinges much of the proper appreciation of the function of organs and their reparative abilities.

The necessity for thorough diagnosis is one of the most self-evident facts connected with the handling of surgical cases. In addition to this, a well grounded knowledge of pathology and especially surgical pathology make one either the surgical scientist or artisan. It is quite important to know what is the condition requiring an operation, or whether an opertions should be performed or not. The proper study of surgery through a systematic and thorough course, after the preliminary preparations have been gone through with is a vital necessity and in the study of surgery, experiments should be practiced upon the cadaver, the lower animals and such other appliances as may be utilized for practical purposes. It is much better that these experiments be made upon the lower animals or upon the cadaver in the laboratory than that they should be practiced upon the living human being. In fact, many of the surgical principles should by all means be undertaken upon such subjects before being undertaken upon a human being. The skill and dexterity in handling tissues may be perfected through operations upon the lower animals which may prove the means ultimately of saving many human lives. I allude especially to many of our abdominal and intestinal operations as well as the operations upon the vascular system, which may be worked out upon the lower animals before being undertaken upon a human being, thereby oft times saving lives that may otherwise have been sacrificed. Not enough work of this sort has been done particularly in our Southern Schools for the perfection of this art. This practice should be more extensively employed and good results in the way of surgical research will be very much encouraged if so carried out. In fact, the experimental surgical work should always be elaborated in the laboratories before being tested upon the human being.

After the completion of such a course of surgical study, if one desires to confine his work to the art of surgery, he should identify himself with some well equipped hospital wherein experience in medicine as well as surgery may be engaged in. The necessity for an extensive study of medicine is vitally important for fear of making one too narrow and seeing everything only through surgical eyes, and the careful study of surgical pathology, studying of tissues and secretions, where the patients are kept under observation, is of the greatest possible value.

After serving through this period, one should then act as an assistant to some well established and progressive surgeon, whose methods he must admire and whose practice he can in a measure at least emulate, though I do not believe that it is well for a young surgeon to endeavor to absolutely imitate any one, be he ever so great.

This hospital experience should extend through a period of about two years going through all of the various phrases of hospital life. After completing this hospital experience, he should identify himself if possible to act as his regular assistant, assisting him in the development of his cases, the selection of his instruments to be used and in a measure directing his technique, assisting in the performance of operations and following the progress of cases throughout their surgical experiences. He should serve for a period of several years in this capacity until he has acquired a maturity of surgical judgment. This, however, comes from a wide experience under proper direction and careful study. During all this time, he should place himself where abundant surgical literature could be obtained and same should be carefully read and digested, making such notes of articles of special interest and keeping careful records of cases that are of special interest or rarity.

He should identify himself with medical associations, both local and national and should participate in the discussions and presentations of interesting cases, giving his views as to the surgical handling of such cases, demonstrating post-operative results. In addition to this, he should visit the various clinics of value throughout the country and study the methods that are practiced by other surgeons. These visits should be made several times each year if possible, selecting such men as are developing new and practical surgicals principles, staying sufficiently long to thoroughly familiarized himself with these methods. If he has carried out, with a degree of thoroughness such a method of training, he would then be what would be considered, a thorough and modern surgeon from a scientific standpoint.

There are yet qualifications, however, which must not be overlooked in order that he may reach this sphere in a manner that would be entirely satisfactory to himself and his clientele. There are at times certain men who under the most careful surgical training could never become surgeons as they were not built out of surgical material. It is useless when such a condition exists to waste time, energy and enthusiasm upon material that is proven incapable of proper developement and when one finds that such is the case, it is much better that he transfer his attention to that field to which he is better adapted. The earlier this is discovered, the better will the subsequent results be.

There is yet another feature that must not be passed without being given serious attention and that is the moral side of the surgeon's life. If one is not morally qualified to handle these conditions, he should under no circumstances engage in the practice of this art, and when I speak of the moral side, I mean that he must have acquired a just and honorable character and abide by it in reference to his patients as much as though it were for himself or his own family.

There is now in certain sections a tendency on the part of some surgeons towards a mercenary spirit. I allude especially to the surgeons whose conscience becomes so lax in reference to so-called nausea of pregnancy, leading them oft times to inducing an abortion among their wealthy clinenteles, when the conditions do not entail the risk to the life of the mother if this pregnancy was allowed to go on uninterfered with. This kind of a conscience becomes oft times very elastic and should have no place in the life of a surgeon. There are times when inducing of abortion is a necessity in order that the life of the mother may be saved. In such cases it would be culpable on the part of the surgeon not to resort to such means after he has exhausted every resources for correcting the condition by other means.

Another practice that is to be greatly deprecated is that of so-called fee splitting. This is an extremely difficult problem to handle, but is certainly a dishonest practice and if a surgeon be dishonest in one particular, he will also be dishonest in others. The surgeon who is willing to stoop to such methods will ultimately be persuaded to operate upon patients when there is no necessity for such an operation and for that reason this is a most condemnable practice and one which we cannot frown upon too severely.

5—Medical Journal—E. W. A. & Co.

There are many other things which may have been added to this brief paper, but I have only touched upon those things which I think important and alluded to a few abuses that we ought as far as possible avoid.

